# 

**Published:** 1887-06-11

**Authors:** 


					CL^
PRICE ONE PENNY. A / u " A/(
No. 37, Vol. II.
June 11th, 1887.
i e8isf ered at tlie General Post Office
as a Newspaper.]
fii
u
Per Annum, 6s. 6d.,post free.
Monthly Parts, 6d.; post free, 7?d.
Ditto per Ann., 7s. 6d., post free.
^   7 V U -r
^^^R^r[5T"TOMKaS'S 'Hospi C&l' ? '..'* ?? !>c and f/ontrick t/nv nihil-
I an Institution, .ifamilp, anti $arocf)ial Journal of
ffiostritals, agpluwg. & all agcncicg for tfie (Hare of tfft g>icfr. Criticism & flietog.

				

